# A role of arginase-1-expressing myeloid cells in cachexia

**DOI:** 10.1186/s40170-025-00396-0

**Published:** 2025-06-05

**Authors:** Apsana Lamsal, Sonja Benedikte Andersen, Unni Nonstad, Natalie Jayne Kurganovs, Richard JE Skipworth, Geir Bjørkøy, Kristine Pettersen

**Affiliations:** 1 Department of Biomedical Laboratory Science, Faculty of Natural Sciences, Norwegian University of Science and Technology, Trondheim, Norway; 2 Centre of Molecular Inflammation Research, Department of Cancer Research and Molecular Medicine, Faculty of Medicine and Health Sciences, Norwegian University of Science and Technology, Trondheim, Norway; 3 Department of Molecular Cell Biology, Institute for Cancer Research, Oslo University Hospital, Montebello, Oslo Norway; 4 Centre for Cancer Cell Reprogramming, Faculty of Medicine, University of Oslo, Oslo, Norway; 5 Institute for Cancer Research, Department of Tumor Biology, Oslo University Hospital, Montebello, Oslo Norway; 6 Clinical Surgery, University of Edinburgh, Royal Infirmary of Edinburgh, Edinburgh, Scotland

**Keywords:** Cancer, Cachexia, Arginine, ARG1, Muscle, Autophagy, Neutrophil, Myeloid-derived suppressor cell, Mitochondria, Mitophagy

## Abstract

**Background:**

Despite decades of efforts to find successful treatment approaches, cachexia remains a major unmet medical need. This condition, that affects patients with diverse underlying conditions, is characterized by severe muscle loss and is associated with reduced quality of life and limited survival. Search for underlying mechanisms that may guide cachexia treatment has mainly evolved around potential atrophy-inducing roles of inflammatory mediators, and in cancer patients, tumor-derived factors. Recently, a new paradigm emerged as it is becoming evident that specific immune cells inhabit atrophic muscle tissue. Arginase 1 (*Arg1*) expression is characteristic of these immune cells. Studies of potential contributions of these immune cells to loss of muscle mass and function is in its infancy, and the contribution of ARG1 to these processes remains elusive.

**Methods:**

Analyses of RNA sequencing data from murine cachexia models and comprehensive, unbiased open approach proteomics analyses of skeletal myotubes was performed. In vitro techniques were employed to evaluate mitochondrial function and capacity in skeletal muscle cells and cardiomyocytes. Functional bioassays were used to measure autophagy activity. ARG1 level in patients’ plasma was evaluated using ELISA, and the association between *ARG1* level and patient survival, across multiple types of cancer, was examined using the online database Kaplan-Meier plotter.

**Results:**

In line with arginine-degrading activity of ARG1, we found signs of arginine restriction in atrophic muscles. In response to arginine restriction, mitochondrial functions and ATP generation was severely compromised in both skeletal muscle cells and in cardiomyocytes. In skeletal muscle cells, arginine restriction enhanced the expression of autophagic proteins, suggesting autophagic degradation of cellular content. Reduction in mitochondria marker TIMM23 supports selective autophagic degradation of mitochondria (mitophagy). In arginine starved cardiomyocytes, mitochondrial dysfunction is accompanied by both increased bulk autophagy and mitophagy. In cancer patients, we found an association between ARG1 expression and accelerated weight loss and reduced survival, further supporting a role of ARG1-producing cells in cachexia pathogenesis.

**Conclusion:**

Together, our findings point to a mechanism for cachexia which depends on expansion of ARG1-expressing myeloid cells, local restriction of arginine, loss of mitochondrial capacity and induced catabolism in skeletal muscle cells and in the heart.

**Supplementary Information:**

The online version contains supplementary material available at 10.1186/s40170-025-00396-0.

## Background

Cachexia is a multifactorial condition that results in a life-threatening degradation of muscle tissue. The condition is a serious clinical consequence of many chronic illnesses at advanced stage, including cancer, chronic heart failure, chronic kidney failure and chronic obstructive pulmonary disease [1]. The pathophysiology of cachexia is complex, but muscle atrophy (of both heart and skeletal muscle) is a central limiting factor for survival and functionality. Although cachexia has been recognized for centuries, and despite numerous suggested cachexia-causing factors, the key underlying elements that can efficiently be targeted in treatment remain to be identified.

Among cancer patients, up to 80% are affected by cachexia [1]. Cancer cachexia is initiated by cancer cells and at present, only curative cancer treatment can successfully reverse the condition. Cachexia is most frequent in aggressive cancers, such as gastrointestinal and lung cancer [2]. Aggressive cancer development is also associated with the recruitment of immune cells that triggers anti-inflammatory responses and suppresses tumor immunity [3]. Although the alterations in immunological components in response to a tumor may have systemic effects, it is still unknown whether the ability of a tumor to cause cachexia may be mediated through immune cells directly, rather than exclusively by soluble signaling factors in circulation.

The muscle microenvironment is a complex constitution of various cell types and extracellular matrix in which also immune cells are important [4]. The level and activity of immune cells in this environment is dynamic. Despite increased understanding of immune cell involvement in general muscle maintenance, our knowledge about the putative contribution of immune cells to cancer cachexia is in an infant stage.

Muscle loss and loss of muscle function in cachexia is associated with abnormal metabolism. The pronounced metabolic derangements that occur in atrophic muscles, include altered metabolism of glucose, lipids, and amino acids [5–7]. One of the most significantly disturbed metabolic pathways in atrophic muscles is the urea cycle [5, 6], including accelerated turnover of urea cycle intermediates. The enzyme responsible for turnover of arginine to ornithine, a key step in the urea cycle, is arginase 1 (ARG1). Interestingly, in addition to high expression of this enzyme in hepatocytes, ARG1 expression is characteristic for some specific immune cells. In mice, various myeloid cells, including M2 macrophages, neutrophils, and myeloid derived suppressor cells (MDSCs) express ARG1, while in humans, expression is restricted to neutrophils and MDSCs [8].

An attention to MDSCs in cachexia research has sparked as these cells are found in large numbers in several mouse models of cachexia, including the 4T1 mammary carcinoma, the CT26 colon adenocarcinoma and the Lewis lung carcinoma (LLC) model [9]. In all these models, increased level of MDSCs in bone marrow and spleen is associated with weight loss [9]. Deyhle et al. (2022) showed, using a pancreatic cancer-induced model of cancer cachexia (KPC), that Ly6G-positive myeloid cells accumulate in muscle tissue of cachectic animals and that Ly6G-depleted animals are partly protected from cachexia [10]. Ly6G is a granulocytic cell marker and can be expressed on both neutrophils and polymorphonuclear (PMN)-MDSC [11], suggesting that more or less mature myeloid cells in the granulocytic branch are important in cachexia development. Following these studies, further characterization of muscle-infiltrating immune cells has been performed. Also here is an expansion of granulocytes, PMN-MDSCs and/or neutrophil-like monocytes evident [12, 13].

We hypothesized that ARG1 expressing myeloid cells cause arginine deficiency in muscle tissue during cachexia and that this leads to compromised mitochondrial capacity and increased catabolic activity. Our findings indicate that ARG1-mediated degradation of intramuscular arginine may be an important mechanism for loss of muscle function in cachexia patients.

## Methods

### Patient material

Heparin plasma samples from oesophagus, gastric or pancreatic cancer patients were obtained from the Edinburgh Cancer Cachexia Cohort (ECCC) Study, Edinburgh, Scotland and divided into 3 groups; healthy controls, cancer patients with < 2% and cancer patients with > 10% self-reported weight loss during the last 6 months prior to sampling, referred to as non-cachectic and cachectic, respectively (Table S1, Additional file 1). Experiments were performed in accordance with the approval from the local ethical committee (Lothian Regional Ethics Committee (REC), Scotland, 06/S1103/75). Written informed consent was received from participants prior to inclusion in the study and the study was performed in conformity with the declaration of Helsinki.

### Cell lines and cell culture

AC16 human cardiomyocytes (Sigma, SCC109) were cultured in DMEM/F12 (Sigma, D6434) supplemented with 12.5% fetal calf serum (Thermo Fischer Scientific, Gibco #10272-106) and 2 mM L-glutamine (Lonza Group, Cat #De-17-605E). C2C12 murine myoblasts (ATCC, CRL-1772) were cultured in DMEM (Sigma, D6429) supplemented with 10% fetal calf serum (Thermo Fischer Scientific, Gibco #10272-106). When differentiating C2C12 cell into myotubes, myoblasts were seeded in conventional medium. Once the culture reached 80–90% confluency, the medium was exchanged to differentiation medium containing growth medium (Sigma D6429), supplemented with 2% horse serum. The medium was replaced with fresh differentiation medium twice during the following six days before myotubes were formed and experiments were initiated. All cells were incubated at 37 °C with 5% CO2.

### Transcriptomic data analysis

Publicly available RNA sequencing data sets of gene expression in muscles from three different models of tumor bearing mice were reanalyzed, as explained below. The three models included allografts of Lewis lung carcinoma (LLC) [14], 4T1 (metastatic breast cancer cells) or C26m2 (a metastatic subline of C26 colon cancer cells) [15]. In brief, 4T1- and C26m2-bearing mice underwent tumor resection following 2–3 weeks of tumor growth. After additional 2–3 weeks, to allow metastases to form following the tumor-resection-relapse approach, the cachectic tibialis anterior was used for RNA isolation and sequencing. For LLC, mice were euthanized following 21 days of tumor growth, and RNA from gastrocnemius tissue was sequenced. Sequencing data are available in the Gene Expression Omnibus database (GEO number GSE152553 and GSE112204).

The transcriptome data for the LLC model were analyzed for differential gene expression using DESeq2 package in R studio [16]. Genes with total count < 5 were filtered out using filterfun function in RStudio and default settings for DESeq2 were used. The presence of differentially expressed genes (DEGs) was determined by comparisons of control and cachexia groups. The cut off log2FC of > ± 2 and *p.*adj < 0.05 was used to define DEGs. For 4T1 and C26m2 models, the log2FC and *p* value provided in the publicly available RNA sequencing datasets were used for further analysis. The same threshold with log2FC > ± 2 and *p.*adj < 0.05 were used to define DEGs in 4T1 and C26m2 models. Enhanced Volcano R package (v1.17) was used to generate volcano plots. Functional enrichment analyses of DEGs were performed to evaluate common biological functions related to DEGs. Gene ontology (GO) functional enrichment analyses for biological process (BP) for DEGs from different groups applicable were performed using the clusterProfiler package from Bioconductor [17]. The ggplot2 package (v3.2.1) was used to visualize the plots.

### Arginine starvation media

During arginine restriction, C2C12 and AC16 cells were grown in SILAC medium lacking L-arginine and L-lysine (Thermo Fisher, 88364 and 11140035, respectively), supplemented with 10% and 12.5% dialyzed fetal bovine serum (Thermo Fisher, 26400044), respectively, 1% non-essential amino acids (Thermo Fischer, 11140035), L-lysine (146.2 mg/L and 91.25 mg/L, respectively, Sigma-Aldrich, L5501), and the indicated concentrations of L-arginine (Sigma-Aldrich, A6969). We modeled arginine deprivation using 0–4 µM and maintained control conditions at concentrations found in standard growth media. While basal circulating arginine levels range from 21 to 137 µM [18], and around 240µM following arginine and citrulline supplementation [19], the exact concentrations available to muscle cells in vivo, especially under atrophic conditions, remain undefined.

### MS analyses

C2C12 cells were differentiated to myotubes (see section *Cell lines and cell culture*), washed twice in PBS and grown further in medium without arginine, limited arginine (4µM) or surplus arginine (400µM). Following 24–48 h, the cells were lysed in lysis buffer: 8 M urea (Merck Millipore, 1084870500) with 4% CHAPS, 100 mM DTT (Sigma, 646563), 1x Complete^®^ protease inhibitor (Roche, 1187350001), 2x phosphatase inhibitor cocktail II (Sigma, P5726) and III (Sigma, P0044). The cells were shaken for 15 min at 4^o^C before centrifugation (15 000 x g, 20 min, 4 °C). Protein concentration was determined at 595 nm using BioRad protein assay dye reagent (Bio-Rad, 500-0006). Each sample (15 µg protein) was added 130 µl 100 mM ammonium bicarbonate. Proteins were reduced and alkylated with DTT (12 mM) for 30 min at 55 °C and alkylated with iodoacetamide (36 mM) for 30 min at room temperature and dark. Protein digestion with 250 ng trypsin at 37 °C overnight was performed before further acidification in acetic acid (0.5%) and desalting in Oasis HLB C18 solid phase extraction according to the manufacturer’s instructions. Peptides were eluted and dried in speedvac and further dissolved in 18 µl 0.1% formic acid. LC-MS/MS was performed on a timsTOF Pro (Bruker Daltonics) connected to a nanoElute (Bruker Daltonics) HPLC. Peptides were separated using a Bruker15 (75 μm*15 cm) column with running buffers A (0.1% formic acid) and B (0.1% formic acid in acetonitrile) with a gradient from 0% B to 37% B for 100 min. The timsTof instrument was operated in the DDA PASEF mode (10 PASEF scans per acquisition cycle) and accumulation and ramp times of 100 millisecond each. The ‘target value’ was set to 20 000. Dynamic exclusion was activated and set to 0.4 min. The quadrupole isolation width was set to 2 Th for m/z < 700 and 3 Th for m/z > 800.

### Proteomics data analysis and bioinformatics analysis

Proteins were quantified by processing MS data using MaxQuant v.2.0.3.0 [20]. The open workflow provided in FragPipe [21] was used to inspect the raw files to determine optimal search criteria and accordingly search parameters were set as follows: enzyme specified as trypsin with maximum two missed cleavages allowed; deamidation of asparagine/glutamine, oxidation of methionine, and protein N-terminal acetylation as variable modifications; precursor and fragment mass tolerance were set to 20 parts per million (PPM). These were imported in MaxQuant which uses m/z and retention time (RT) values to align each run against each other sample with a minute window match-between-run function and 20 min overall sliding window using a clustering-based technique. These were further queried against the mouse proteome including isoforms downloaded from Uniprot in 2021 along with MaxQuant’s internal contaminants database using Andromeda built into MaxQuant. Both protein and peptide identification false discovery rate (FDR) were set to 1%, only unique peptides with high confidence were used for final protein group identification. Peak abundances were extracted by integrating the area under the peak curve. Each protein group abundance was normalized by the total abundance of all identified peptides for each run and protein by calculated median summing all unique and razor peptide-ion abundances for each protein using label-free quantification (LFQ) algorithm [22] with minimum peptides ≥ 1. LFQ values for all samples were log-transformed with base 2. A correlation heatmap using R package pheatmap [23] created using these transformed LFQ values and an outlier was removed. The rest of the values representing each condition were subjected to two-sided Student’s t-tests as implemented in R in order to check the consistency of change. The amount of change was estimated by subtracting the median of these values representing each group (log2FC). Further, to estimate the false-discovery rate (FDR), the t-test *p*-values were corrected using the Benjamini-Hochberg procedure. Differentially expressed (DE) protein groups were identified at FDR < 0.1 and absolute log2FC > 0.5. The DE quantified only in one group were checked if their coefficient-of-variation of log2FC was within 5%. The Uniprot accession IDs of these DE were mapped to a volcano-plot using R package ggplot2 [24]. Principal component analysis (PCA) was performed using Pearson correlation via online tool Morpheus [25]. Volcano plots represented in the figures were drawn using the EnhancedVolcano R package (v 1.17), and the cut off was set to log2 median change ranging from ± 0.5 and the corrected t-test *p*-value of < 0.05. Log2 median change is represented as log2FC throughout the article.

### LDH sequestration assay

LDH sequestration was determined as described previously [26, 27] with slight modifications. AC16 cells were seeded (125 000 cells per well) in six-well plates (as triplicates in 4 independent experiment) and grown to 60–70% confluency. The cells were washed twice in PBS and transferred to medium without or with arginine (400µM) (see section *Arginine starvation media*) and Bafilomycin A1 (Santa Cruz Biotechnology, sc-201550 A; 100 nM) as indicated for 24 h. Cells were harvested in trypsin-EDTA (0.25%) and collected in complete medium. After centrifugation at 400 × g for 5 min at 4 °C, the supernatant was aspirated, and cells were resuspended in 400 µl of isotonic sucrose (10%). To selectively disrupt the plasma membrane, cells were subjected to an electric pulse (2000 V and 25 microfarads in a 1 × 1 × 5-cm electrode chamber) with the homemade apparatus and subsequently mixed with 400 µl of phosphate-buffered sucrose (100 mM sodium monophosphate, 2 mM DTT, 2 mM EDTA, and 1.75% sucrose, pH 7.5) to a total volume of ~ 750 µl. 150 µl of cell disruptate was removed for total LDH measurements (“LDHTotal”) and was stored overnight at − 80 °C. 550 µl of cell disruptate was resuspended in 900 µl of resuspension buffer (50 mM sodium monophosphate, 1 mM EDTA, 1 mM DTT) supplemented with 0.5% BSA and 0.01% Tween 20. Cell corpses, containing autophagic vacuoles, were sedimented by centrifugation at 18 000 × g for 45 min at 4 °C. Supernatant was aspirated, and the pellet (“LDHSediment”) was stored overnight at − 80 °C. The following day, both LDHTotal and LDHSediment were diluted in resuspension buffer supplemented with Triton X-405 (Sigma-Aldrich, X-405) to a final concentration of 1%. After a short spin (18 000 × g for 10 min at 4 °C), the enzymatic activity of LDH in LDHTotal and LDHSediment was measured as described previously [26] using homemade reagents, mixing 4 volumes of 65 mM imidazole (pH 7.5), 0.75 mM pyruvate with one volume of 65 mM imidazol (pH 7.5), 1.8 mM NADH. LDH sequestration activity was calculated as percentage of sedimentable LDH in experimentally treated cells minus percentage of sedimentable LDH in untreated cells (background), divided by the incubation time with BafilomycinA1. For a detailed description of the protocol, see [26]. A similar approach was used to perform the LDH sequestration assay in C2C12 cells. Briefly, 125,000 cells were seeded per well in six-well plates (in triplicates, across two independent experiments). Upon reaching approximately 80% confluency, the cells were switched to differentiation medium. After 4–6 days in differentiation conditions, when differentiated myotubes predominated, the cells were subjected to arginine starvation for 24 and 48 h. LDH release was then assessed as described above.

### Generation of stable cell lines expressing inducible mito-mKeima

AC16 cells were grown in 6 well cell culture plates until about 80% confluency. Lentiviral constructs, a kind gift from Dr. Lisa Frankel (Danish Cancer Society Research Center), were used to generate the viruses as in [28]. Cells were incubated with or without 500 µl of virus, in addition to 8 µg/ml polybrene, and 20 h after infection they were washed with DPBS and given fresh culture medium. The cells were split 48 h after starting the infection, and stably transduced cells were selected by maintaining the cells in 2 µg/ml puromycin (Sigma-Aldrich, P8833). To turn on the expression of the mKeima variants, cells were treated with 250 ng/ml doxycycline (Sigma-Aldrich, D9891) and the activity of the constructs were validated by flow cytometry as in [28].

### Assessing autophagic flux by flow cytometry

AC16-mt-mKeima cells were seeded in 6 well plates with regular DMEM containing 250 ng/ml doxycycline and grown for 2 days to 40% confluency. The cells were then grown in SILAC-medium with 400 µM or 0 µM arginine for 48 h (see section *Arginine starvation media*), washed twice with DPBS and trypsinized. The trypsin was inactivated using DPBS with 40% dialyzed FBS (Gibco, 26400044), and cells were sedimented by centrifugation at 400 x g for 5 min at 4 °C. Cell pellets were resuspended in 150–400 µl FACS buffer (DPBS with 2% FCS and 0.2 mM EDTA) and stained with Fixable Blue Dead Cell Stain UV (Invitrogen, L34961; 1:1000 in DPBS) for 30 min on ice. The cells were washed twice, resuspended in FACS buffer, and analyzed on a BD LSR II flow cytometer in biological and technical triplicates recording 200 000 events per sample. mKeima was excited in each cell by 407 nm and 561 nm lasers, and a 610/20 bandpass filter and 600 nm long pass dichroic filter were used to acquire the emission. The fcs files were analyzed in the FlowJo10.2 software. Cell gates were set after the exclusion of doublets and dead cells, followed by gating of mKeima-positive cells (Fig.S1, Additional file 2). Designated live, single cells positive for mKeima were plotted for the fluorescent signals obtained from excitation with the 407 nm laser versus the 561 nm laser. For downstream ratiometric analyses, the fluorescence intensity obtained for each cell from each sample with the 561 nm laser was divided by that obtained with the 407 nm laser, using the “Derive Parameters” function in FlowJo, and the ratio values were plotted as histograms for each treatment condition, as in [28]. Median ratio value from each sample was normalized to that obtained in the sample from control cells, where the median ratio value from control cells was set to 1.

### XF96 seahorse test of mitochondrial function (Mito stress test)

AC16 cells (3000 cells in 80 µl/well) or C2C12 cells (3000 and 5000 cells for myoblasts and myotube experiments, respectively) were seeded in XF96 polystyrene cell culture microplates that had been pre-treated with Cell-Tak Cell and Tissue adhesive (Fisher Scientific, CB-40240), according to the manufacturer’s instructions. The next day, the growth media was removed from half of the plate, the cells were washed twice in PBS (100 µl/well), and they were given medium (100 µl/well) without arginine or with varying concentrations of arginine (see section *Arginine starvation media*). The following day, the remaining half of the plate was treated as described above, while the other half of the plate containing cells already grown in varying arginine levels for 24 h were left untouched. For C2C12 cells differentiated into myotubes a 6-day differentiation procedure preceded arginine starvation (see section *Cell lines and cell culture*). After yet another 24 h, all cells were washed twice (100 µl/well) and incubated at 37 °C in a CO2-free incubator in serum-free Seahorse XF Base Medium (Agilent, 102353-100) supplemented with 10 mM glucose, 10 mM pyruvate and 2 mM (for AC16) and 4mM (for C2C12) L-glutamine and with a pH adjusted to 7.4 at 37^o^C (180 µl/well). After about 1 h of incubation, the plate was transferred to the Seahorse XF96 analyzer and basal oxygen consumption rate (OCR) was measured. Then, the OCR was monitored after injections of oligomycin (Sigma-Aldrich, O4876; 1 µM for AC16 and 1.2 µM for C2C12), carbonyl cyanide 4-(trifluoromethoxy)phenylhydrazone (FCCP, Sigma-Aldrich, C2920; 1 µM for AC16, 2 µM for C2C12 myoblasts and 2.5 µM for C2C12 myotubes) and the combination of antimycin A (AA; Sigma-Aldrich, A8674; 1 µM) and rotenone (Sigma-Aldrich, R8875; 1 µM). After the analysis, all medium was removed, the plate was sealed and stored at -80^o^C. For normalization, nuclear DNA was stained using CyQUANT cell proliferation assay (Invitrogen, C7026) according to the manufacturer’s instructions. Fluorescence was measured on a Spark plate reader (Tecan) with excitation at 485 nm, emission at 520 nm and bandwidth of 10 nm. The experiments were performed 4 times for AC16, 3 times for C2C12 myoblasts and 5 times for C2C12 myotubes, each time using 11 or 12 wells per condition.

### Elisa

Arginase 1 level in plasma was determined using a Human ARG1 elisa (Invitrogen, BMS2216) according to the manufacturers protocol.

### Use of public databases

Kaplan-Meier plotter [29] is an online database that utilizes data from multiple cDNA microarrays for examining prognostic markers in several cancer types. The association between overall survival (OS) and expression of *ARG1* was analyzed in colon cancer, gastric cancer, pancreatic ductal adenocarcinoma and lung adenocarcinoma patients. The auto-selected best cutoff value was chosen to divide patients into high expressing and low expressing groups.

### Statistical analysis

Statistics for RNA sequencing data are described under Transcriptomic data analysis, and MS analyses are described under proteomics data analysis and bioinformatics analysis. All other statistical analyses were performed in GraphPad Prism 10 and are specified in the appropriate figure texts. Values are expressed as mean ± standard error of the mean (SEM) unless otherwise stated. *p*-value < 0.05 was considered statistically significant and is labeled with *, *p* < 0.01 is labeled with **, *p* < 0.001 is labeled with *** and *p* < 0.0001 is labeled with ****.

## Results

### Elevated expression of neutrophil and MDSC markers in atrophic muscles

**Fig. 1:**
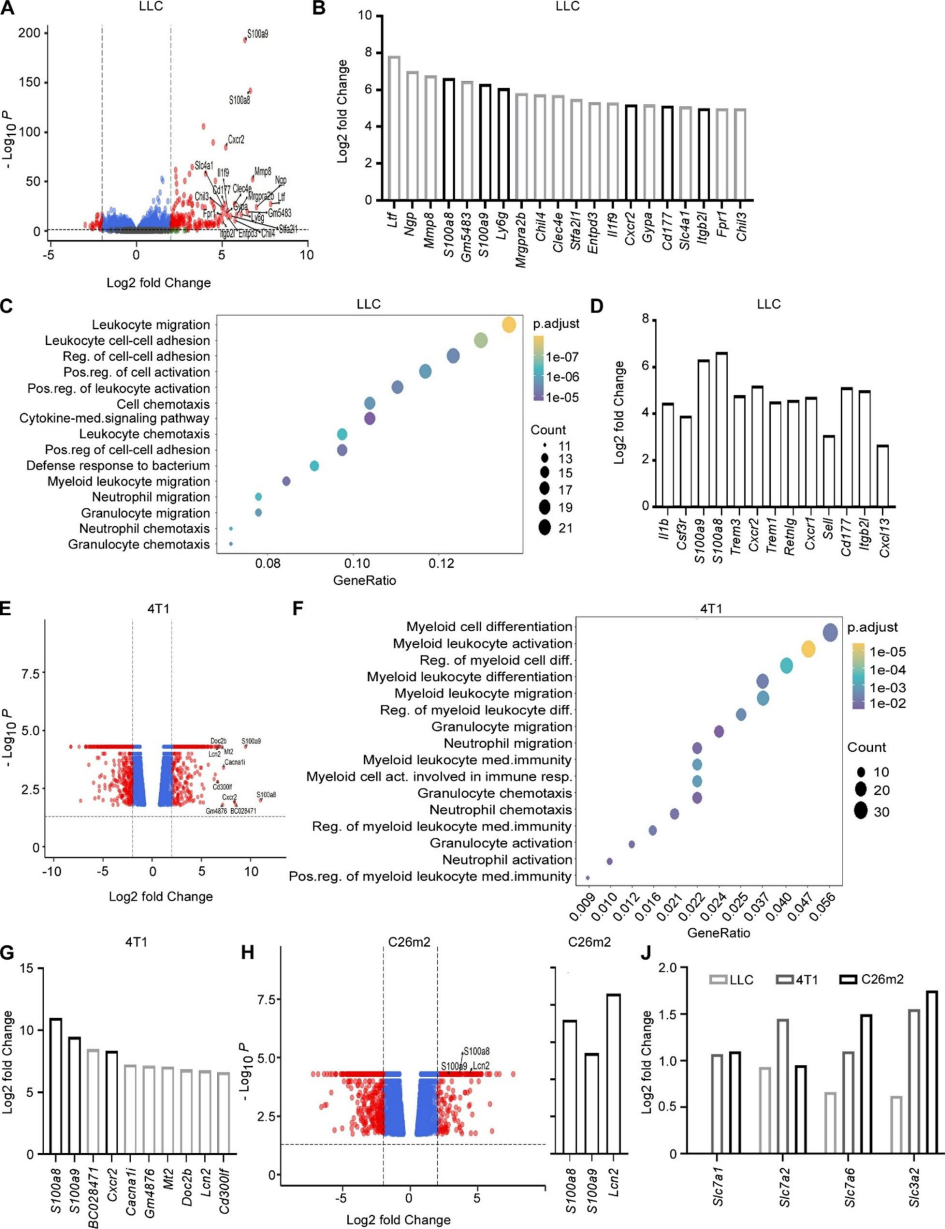
Markers of neutrophils and MDSCs are enriched in atrophic muscle from mice. (**A**) Volcano plots showing differentially expressed genes in muscles from LLC-bearing mice vs. healthy mice. Red points represent genes with log2FC within the cut-off > ± 2 and adjusted *p*-value < 0.05. (**B**) Top 20 most upregulated transcripts in muscles from LLC-bearing mice vs. healthy mice. (**C**) Gene Ontology (GO) analysis of the most enriched biological processes (BP) associated with upregulated genes in muscles from LLC-bearing mice vs. healthy mice (log2FC > 2). (**D**) Upregulated transcripts in LLC-bearing mice vs. healthy mice (log2FC > 2, adjusted *p*-value < 0.05) that are associated with the biological processes “myeloid leukocyte migration”, “neutrophil migration”, “granulocyte migration”, “neutrophil chemotaxis”, and “granulocyte chemotaxis”. (**E**) As in A, but from 4T1-bearing mice. (**F**) GO enrichment-defined biological processes identified by the terms “myeloid”, “neutrophil” or “granulocyte” associated with the upregulated transcripts in muscles from 4T1-bearing mice vs. healthy mice (log2FC > 2). (**G**) Top 10 most upregulated transcripts in muscles from 4T1-bearing mice vs. healthy mice. (**H**) As in A, but from C26m2-bearing mice. (**I**) Upregulated *S100a8*,* S100a9* and *Lcn2* transcripts in C26m2-bearing mice vs. healthy mice. (**J**) Expression of transcripts encoding plasma membrane transporters that accept arginine as a substrate, in atrophic muscles from Lewis lung carcinoma (LLC)-, 4T1, and C26 tumor-bearing mice vs. healthy murine muscles (Log2FC)

Recent studies support an expansion of myeloid immune cells in cachexia [9, 10, 12]. To further investigate this, we reanalyzed a publicly available RNA sequencing data set from muscles of LLC-bearing cachectic mice [14]. In atrophic muscles, the expression was significantly higher for 165 transcripts and lower for 24 transcripts, as compared to muscles from healthy mice (log2FoldChange (FC) > ± 2; adjusted *p*-value < 0.05) (Fig. 1a, Additional file 3). When looking at all significantly elevated transcripts in atrophic muscles from LLC-bearing mice, several of the transcripts associated with myeloid cells, and granulocytes in particular, rank among the top 20 most differentially expressed transcripts (Fig. 1b). This included *S100a8* and *S100a9*, whose corresponding proteins dimerize to form calprotectin [30]. These proteins can be produced by myeloid cells, and account for about half the protein content in neutrophils, and to a lesser extent in reactive tissue macrophages [31]. In addition, the granulocyte markers *Ly6g*, *Cxcr2 and Cd177* [32], as well as *Itgb2l* which together with *Cd177* is associated with neutrophil migration [33], appear among the most enriched transcripts in the atrophic muscle. Performing gene ontology (GO) enrichment analysis for biological processes (BP) associated with the highly expressed genes in atrophic muscles, we found that BPs like “myeloid leukocyte migration”, “neutrophil migration”, “granulocyte migration”, “neutrophil chemotaxis”, and “granulocyte chemotaxis” were significantly enriched upon cachexia. (Fig. 1c, Additional file 4). Although the specific transcripts associated with these processes (Fig. 1d) are related to neutrophils, the phenotypic overlap between mature neutrophils and the more heterogeneous population of MDSC, particularly GMN-MDSC, makes it difficult to fully distinguish between these populations of cells based on RNA data alone. In examples, *S100a8* and *S100a9*, which are the most elevated transcripts in atrophic muscles, are also characteristic markers for MDSCs [34].

To examine whether markers for neutrophils and/or MDSCs could also be detected in atrophic muscles from other murine models, we reanalyzed a publicly available RNA sequencing data set from muscles of 4T1-bearing cachectic mice [15]. In this model, 650 transcripts had significantly higher, while 712 transcripts had significantly lower expression in atrophic muscle compared to healthy muscles (log2FC > ± 2; adjusted *p*-value < 0.05) (Fig. 1e, Additional file 5). When searching GO enrichment-defined BPs associated with the elevated transcripts for the search terms “myeloid” “neutrophil” or “granulocyte”, we found that the same neutrophil and granulocyte-associated processes detected in the LLC model also was enhanced in the 4T1 model. In addition, several other processes, within these search terms, were identified (Fig. 1f, Additional file 6). Strikingly, when examining all differentially expressed transcripts in atrophic muscles in the 4T1 model, we found that, like in the LLC model, *S100a8* and *S100a9* were among the most differentially expressed transcripts (Fig. 1g). In addition, *Cxcr2* were among the top 10 differentially expressed transcripts, as well as *Lcn2* (known as neutrophil gelatinase-associated lipocalin) (Fig. 1g). This further supports that increased neutrophil/MDSC markers is associated with cancer cachexia.

To further address this, we examined whether neutrophil- and MDSC-associated transcripts also was elevated in atrophic muscles in a third mouse model. We reanalyzed a publicly available RNA sequencing dataset from muscles of C26m2-bearing cachectic mice (a metastatic subclone of C26) [15]. Among the 416 transcripts with higher expression in atrophic muscles compared to healthy muscles (log2FC > ± 2; adjusted *p*-value < 0.05) (Fig. 1h, Additional file 7), *S100a8*, *S100a9* and *Lnc2* were identified (Fig. 1i). Together with other recent studies [9, 10, 12], our findings suggest that the expansion of neutrophils and/or MDSCs are highly stimulated in atrophic muscles and that this event is not restricted to single animal models.

One characteristic of neutrophils and MDSCs is the expression of ARG1 [8]. ARG1 converts arginine to ornithine and urea as a key step in the urea cycle and these immune cells can consequently cause restriction of arginine in their proximity [35]. However, since ARG1 in neutrophils is primarily synthesized during their myelocyte and metamyelocyte stages in the bone marrow [36]), we did not expect to detect high levels of ARG1 mRNA in peripheral tissues such as muscle. In line with this, ARG1 mRNA levels were below detection in the 4T1 and C26m2 models, and only marginally detectable in the LLC model.

Given the known ability of ARG1-expressing cells to deplete arginine, we next asked whether signs of an arginine depleted microenvironment could be detected in atrophic muscles. Since arginine restriction may cause a compensatory upregulation of arginine uptake channels, we examined whether plasma membrane transporters that accept arginine as a substrate [37] were affected in atrophic muscles. We found that in two of the three models (4T1 and C26m2) the level of Slc7a1 (also known as Cat-1) was increased, and in all three models the Slc7a2 (also known as Cat-2) level was increased in atrophic muscles (Fig. 1j). In addition, the level of Slc7a6 and Slc3a2, that together form a heterodimeric arginine transporter [37], was increased in atrophic muscles in all three models (Fig. 1j). This suggests that arginine restriction occurs in muscle tissue during cachexia.

### Arginine restriction causes increased expression of autophagic proteins in muscle cells

**Fig. 2:**
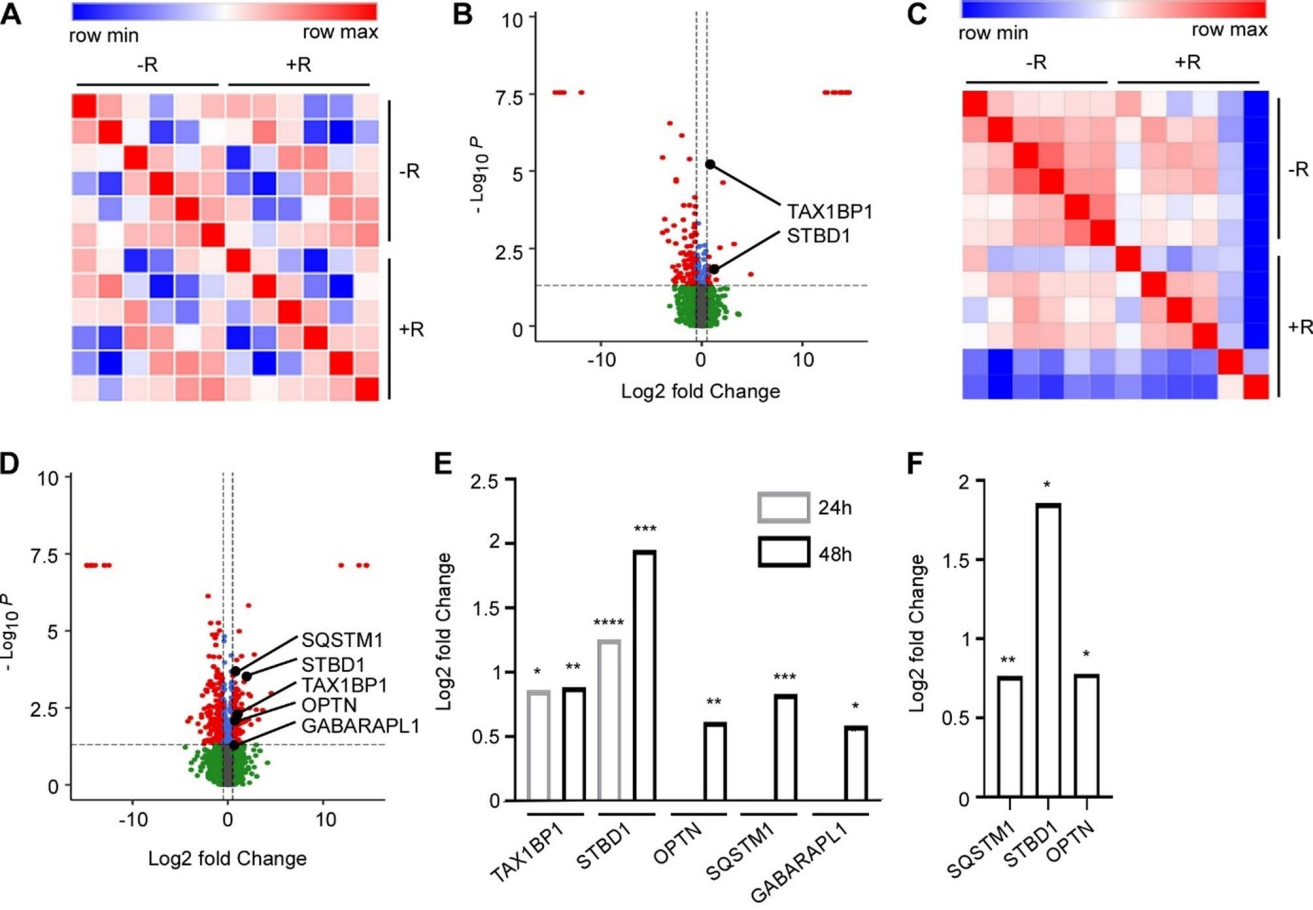
The level of autophagic proteins is increased in arginine-starved muscle cells. (**A**) Pearson correlation matrix of the 4720 proteins detected in mass spectrometry (MS) analysis of C2C12 myotubes grown in medium without arginine or with arginine (400µM) for 24 h (-R and + R, respectively). (**B**) Volcano plots showing differentially expressed proteins in C2C12 myotubes grown without arginine vs. with arginine (400µM) for 24 h. Red points represent proteins with log2FC > ± 0.5 and adjusted *p*-value < 0.05. Autophagic proteins are pointed out. **C-D**) As A and B, respectively, but cells are grown for 48 h. **E-F**) Level of detected autophagic proteins in C2C12 myotubes grown without arginine vs. with arginine (400µM) for 24 h and 48 h, only positive fold change and detected proteins at specified time points shown (**E**) and in C2C12 myotubes grown in minimal arginine (4µM) vs. surplus arginine (400µM) for 48 h (**F**). Statistical significance was evaluated using Student’s t-test corrected using Benjamini-Hochberg. *p < 0.05, ** *p* < 0.01, *** *p* < 0.001 and **** *p* < 0.0001

We, and others, have shown that acceleration of autophagy is important in the pathogenesis of cachexia [38–41]. Amino acid starvation is a strong inducer of autophagy. We have recently shown that arginine restriction is sufficient to potently increase autophagy activity in breast cancer cells [42]. We therefore examined whether restricted availability of arginine will accelerate autophagy also in skeletal muscle cells. To test this, we subjected differentiated C2C12 myotubes to medium without arginine, with limited arginine or with arginine surplus and analyzed for a putative increase in autophagic proteins in response to arginine restriction by mass spectrometry (LC-MS/MS). First, we examined the proteins in C2C12 myotubes grown for 24 h in arginine surplus or complete arginine depletion. Pearson correlation matrix of the 4720 detected proteins showed some variability between biological replicates within each condition. This likely reflects that changes were limited to a specific subset of proteins, rather than broad alterations across the proteome (Fig. 2a). Of these proteins, 102 had lower level and only 34 had higher level in arginine starved cells compared to cells grown in surplus arginine (log2FC > ± 0.5; adjusted *p*-value < 0.05) (Fig. 2b, Additional file 8). When manually inspecting the proteins with elevated level for selective autophagy receptors (using [43] as a guideline) and ATG8 family members, we identified 2 proteins: the selective autophagy receptors TAX1BP1 and STBD1 (Fig. 2b). TAX1BP1 is involved in several types of selective autophagy, including aggrephagy and mitophagy (autophagic degradation of protein aggregates and mitochondria, respectively), while STBD1 has been shown to facilitate the autophagy degradation of glycogen (glycophagy) [43]. Following 48 h of arginine starvation, we also find some variability among biological replicates. This is consistent with the observation that only a specific group of proteins undergo changes, rather than widespread shifts in overall protein expression (Fig. 2c). At this time point, 276 proteins had lower level, and 174 proteins had higher level in arginine starved cells compared to cells grown in surplus of arginine (log2FC > ± 0.5; adjusted *p*-value < 0.05) (Fig. 2d, Additional file 9). Among the proteins with elevated level, 5 autophagic proteins could be detected. These included the autophagy receptors SQSTM1/p62 and OPTN, as well as the ATG8-family member GABARAPL1 (Fig. 2d-e). In addition, elevated expression of TAX1BP1 and STBD1 were maintained or increased as compared to 24 h arginine starvation, respectively (Fig. 2d-e). Notably, when the cells were provided with low levels of arginine, a similar effect on autophagy receptors were observed as for the complete removal of arginine: following 48 h incubation in low arginine medium, the level of the autophagy receptors SQSTM1, STBD1 and OPTN were significantly increased compared to cell grown in arginine surplus (Fig. 2f). However, no significant difference in TAX1BP1 nor GABARAPL1 level was detected.

Elevated expression of autophagic proteins indicates altered autophagic activity. To examine whether arginine starvation increases autophagic activity, we examined the autophagy flux in arginine-starved C2C12 myotubes using a lactate dehydrogenase (LDH) sequestration assay [27]. However, no increased sequestration of LDH could be detected in arginine restricted C2C12 myotubes (Fig.S2, Additional file 10), suggesting that the bulk autophagy process was not accelerated despite increased expression of autophagic proteins. These data indicate that selective autophagy processes might rather be engaged in response to insufficient arginine availability.

### Arginine restriction impairs mitochondrial functions in muscle cells

**Fig. 3:**
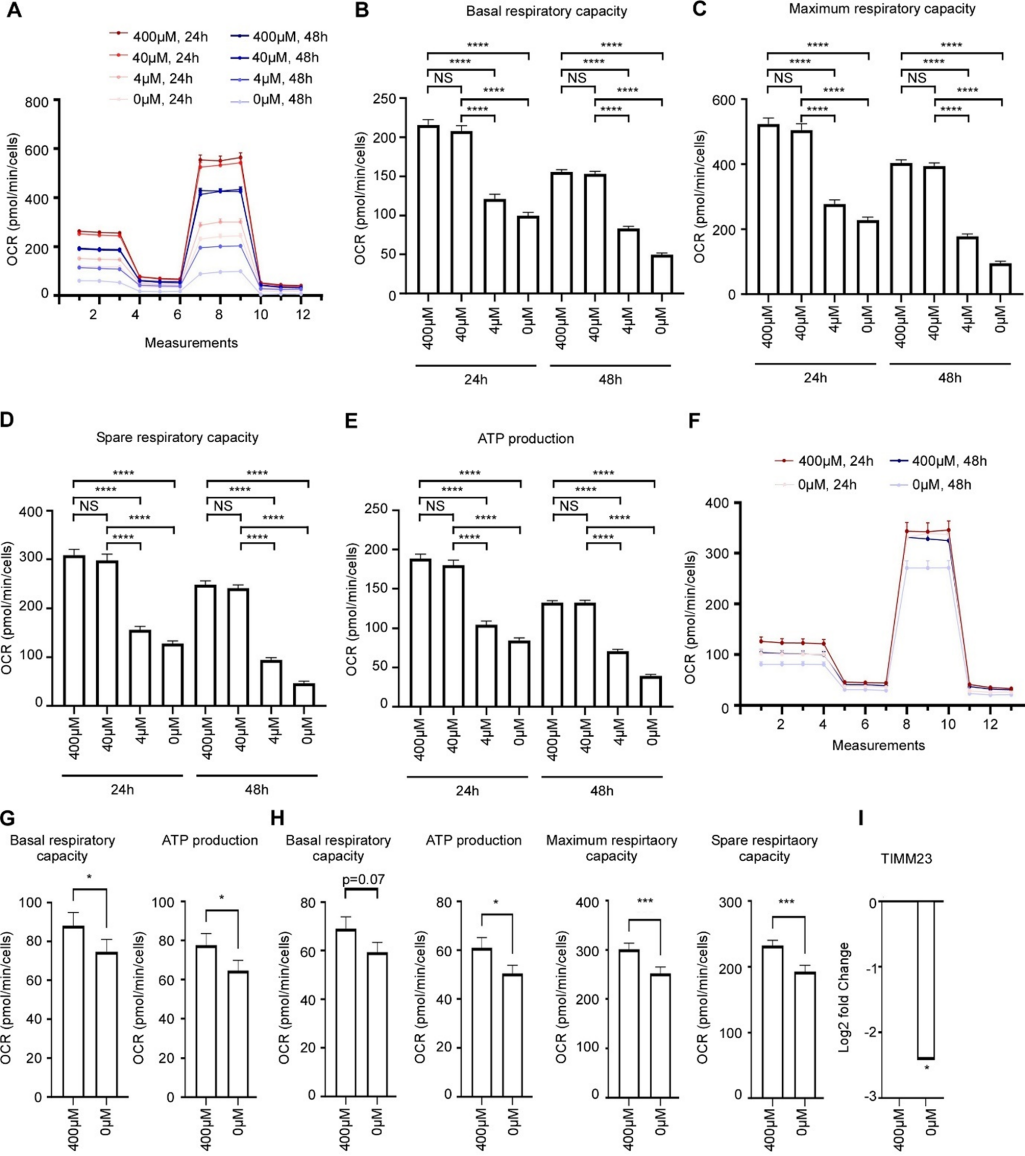
Mitochondrial functions are impaired in arginine-starved muscle cells. **A**) Measurement of mitochondrial function in arginine restricted C2C12 myoblasts, as indicated, using a Seahorse XF96 Analyzer (*n* = 3). Data are shown as mean +/-SEM. B-E) Basal respiratory capacity (**B**), Maximum respiratory capacity (**C**), Spare respiratory capacity (**D**), and ATP production (**E**) in arginine restricted C2C12 myoblasts (*n* = 3). Data are shown as mean +/-SEM. Statistical significance was evaluated using one-way ANOVA followed by Dunnet’s multiple comparisons test. **F**) Measurement of mitochondrial function in arginine restricted C2C12 myotubes, as indicated, using a Seahorse XF96 Analyzer (*n* = 5). **G**) Basal respiratory capacity and ATP production in C2C12 myotubes grown in media containing 400µM or 0µM arginine for 24 h. **H**) Basal respiratory capacity, ATP production, Maximum respiratory capacity, and Spare respiratory capacity in C2C12 myotubes grown in media containing 400µM or 0µM arginine for 48 h (*n* = 5). Statistical significance was evaluated using Mann-Whitney test. **I**) Level of TIMM23 protein in C2C12 myotubes grown in media containing 400µM or 0µM arginine for 48 h. Statistical significance was evaluated using Student’s t-test. NS = not significant, *p < 0.05, ** *p* < 0.01, *** *p* < 0.001 and **** *p* < 0.0001

We [42] and others [44] have shown that Arginine starvation compromise mitochondrial activities and functions in cancer cells. In cancer cachexia, altered muscle mitochondrial functions contribute to muscle wasting [45]. To analyze the impact of arginine restriction on mitochondrial functions in muscle cells, we performed mitochondrial metabolic assessment of C2C12 myoblasts and myotubes using a Seahorse XF96 Analyzer. Growing C2C12 myoblasts in medium without arginine or with limited arginine (4 µM) significantly affected all measured parameters related to the mitochondrial function already after 24 h compared to cells cultured in standard growth medium (400 µM arginine), as well as physiological plasma concentrations [18] of arginine (40µM). Specifically, basal respiration and the ability to perform oxidative respiration under stress (maximum respiration and spare respiratory capacity) were severely compromised (Fig. 3a-d) and ATP production weakened in response to arginine restriction (Fig. 3e). After 48 h of arginine restriction, the effects were even more clear on all parameters (Fig. 3a-e).

Arginine restriction had similar effects on mitochondrial functions in C2C12 myotubes (Fig. 3f-h), yet more modest. In these differentiated skeletal muscle cells, basal respiration and ATP production were affected after 24 h starvation (Fig. 3f-g). Following 48 h arginine restriction, maximum respiratory capacity and spare respiratory capacity also declined (Fig. 3f and h). This shows that both myoblasts and differentiated muscle cells exposed to an arginine restricted environment are unable to maintain normal mitochondrial functions and energy generation. Declining mitochondrial functions may be important for both muscle loss and muscle weakness commonly experienced by cachectic patients.

Damaged or unfunctional mitochondria can be degraded by autophagy in a process called mitophagy [46]. We found that arginine restriction caused declining mitochondrial function and increased expression of autophagic proteins, including proteins involved in mitophagy. We therefore examined whether arginine starvation was associated with increased mitochondrial turnover. For this, we quantified the protein level of the inner mitochondrial membrane protein TIMM23, which serves as an indicator for mitophagy [47, 48]. From LC-MS/MS analysis of arginine starved C2C12 myotubes (48 h) it was clear that the level of TIMM23 protein significantly declined compared to un-starved C2C12 myotubes (Fig. 3i). Since declining level of TIMM23 protein could occur because of either increased mitochondrial degradation or reduced mitochondrial biogenesis, we searched for common mitochondrial biogenesis markers in the MS data. No alteration in the level of the mitochondrial biogenesis markers TFAM and TFB1M [49] were found, neither after 24–48 h of arginine starvation, suggesting that mitochondrial biosynthesis was not significantly affected. Together these data imply that reduced mitochondrial function is associated with reduced mitochondrial mass in arginine restricted skeletal muscle cells.

### Arginine restriction causes loss of mitochondrial capacity and increased mitochondrial turnover in cardiomyocytes

**Fig. 4:**
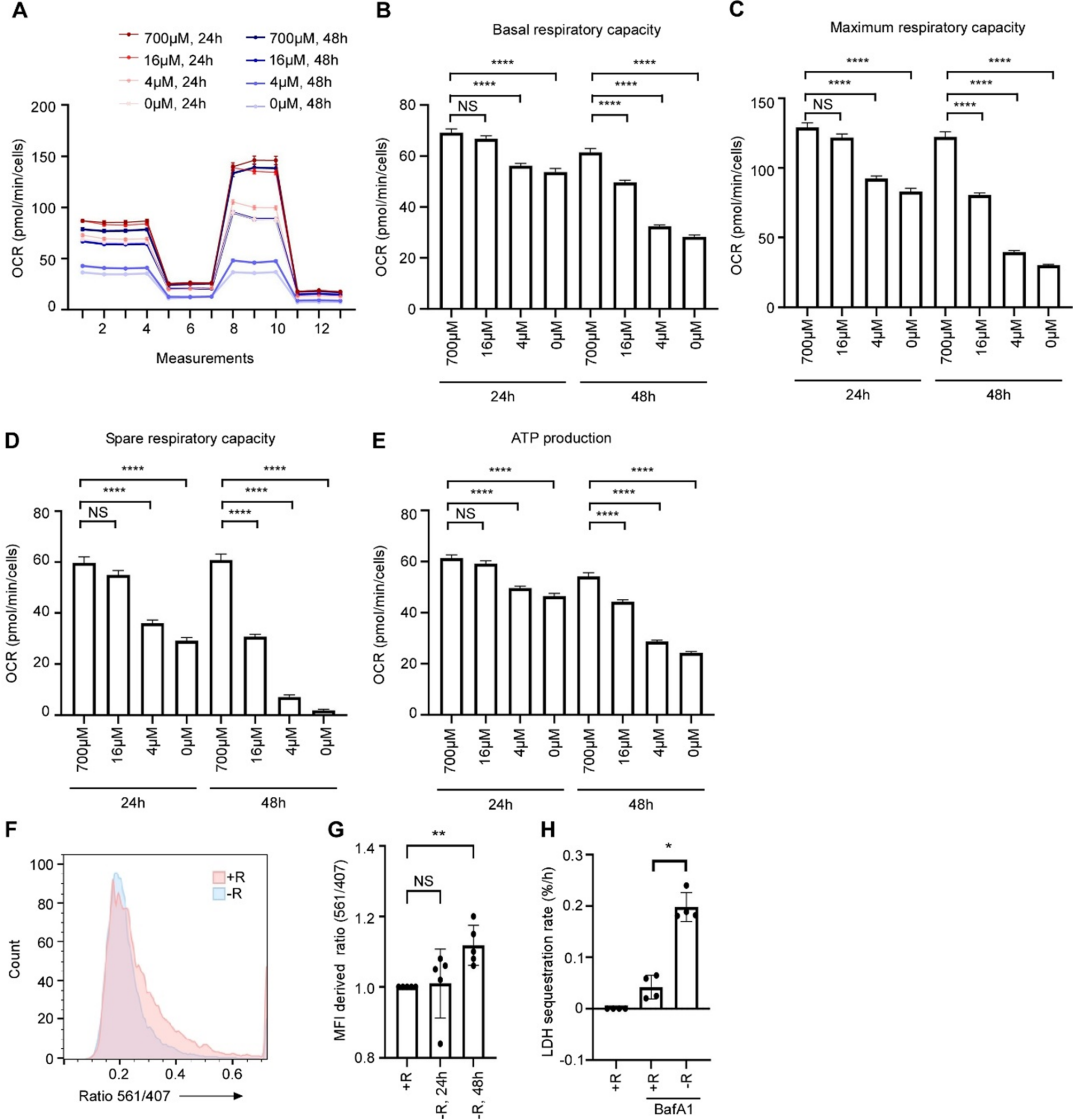
Mitochondrial functions are impaired and mitophagy accelerated in arginine-starved cardiomyocytes. **A**) Measurement of mitochondrial function in arginine restricted AC16 cardiomyocytes, as indicated, using a Seahorse XF96 Analyzer (*n* = 4). Data are shown as mean +/-SEM. B-E) Basal respiratory capacity (**B**), Maximum respiratory capacity (**C**), Spare respiratory capacity (**D**), and ATP production (**E**) in arginine restricted AC16 cardiomyocytes (*n* = 4). Data are shown as mean +/-SEM. Statistical significance was evaluated after log-transformation, using one-way ANOVA followed by Dunnet’s multiple comparisons test. **F**) AC16 cardiomyocytes expressing mito-mKeima grown with 400µM arginine (+ R) or no arginine (-R) (48 h). Representative histograms of the signal ratio by excitation at 561 and 407 nm are shown. **G**) Average median ratio for signals by excitation at 561 and 407 nm in AC16 cardiomyocytes expressing mito-mKeima grown in 400 µM arginine (+ R) or without arginine (-R) for 24 and 48 h (*n* = 5). Data are shown as mean +/-SEM. Statistical significance was evaluated using Kruskal-Wallis followed by Dunn’s multiple comparisons test. H) Lactate dehydrogenase (LDH) sequestration determined in AC16 cardiomyocytes grown in medium with arginine (700µM) or without arginine (+ R and -R, respectively), +/-Bafilomycin A1 (BafA1, 100nM) for 24 h, using an LDH sequestration assay [26] (*n* = 4). Statistical significance was evaluated using Mann-Whitneys test. NS = not significant, *p < 0.05, ** *p* < 0.01, *** *p* < 0.001 and **** *p* < 0.0001

Cardiac atrophy is a pronounced and life-threatening feature of cachexia. It was recently discovered that granulocytic cells, including PMN-MDSCs accumulate in the heart during cachexia [12]. Given that these cells express ARG1, we asked whether arginine restriction could affect mitochondrial functions and autophagy in cardiomyocytes. To assess the effects of arginine restriction on mitochondria, we performed mitochondrial metabolic assessment of AC16 cardiomyocytes using the Seahorse XF96 Analyzer. When depriving the cardiomyocytes of arginine (complete starvation) or when supply minimal arginine (4µM), basal, maximum, and spare respiratory capacity, as well as ATP production was severely reduced (Fig. 4a-e). The effect was most defined following 48 h, however, 24 h arginine restriction also caused a clear effect. Even growing the cells in medium containing 16µM arginine for 48 h compromised all mitochondrial parameters in cardiomyocytes compared to growing the cells in full growth media with arginine surplus.

Given the severely compromised mitochondrial functions in arginine starved cardiomyocytes, we asked whether also mitophagy was accelerated. To examine this, we generated mito-mKeima-expressing AC16 cells. In these cells mKeima is fused to the mitochondrial matrix-targeting presequence of COX VIII and can thereby be used to measure acidification of mitochondria (i.e., mitophagy). Consistent with accelerated mitophagy, 48 h arginine starvation caused a shift of mitochondria to the acidic compartment (Fig. 4f-g). To explore whether arginine restriction also induced bulk autophagy in AC16 cardiomyocytes we used the LDH sequestration assay. Unlike the effect in C2C12 myotubes, arginine restriction significantly boosted the bulk autophagy flux in cardiomyocytes (Fig. 4h). Together, these results show that when arginine availability decline, mitochondrial functions in cardiomyocytes are compromised, and mitophagy and bulk autophagy, is increased.

### Arginase 1 expression is associated with cachexia and poor survival in cancer patients

**Fig. 5:**
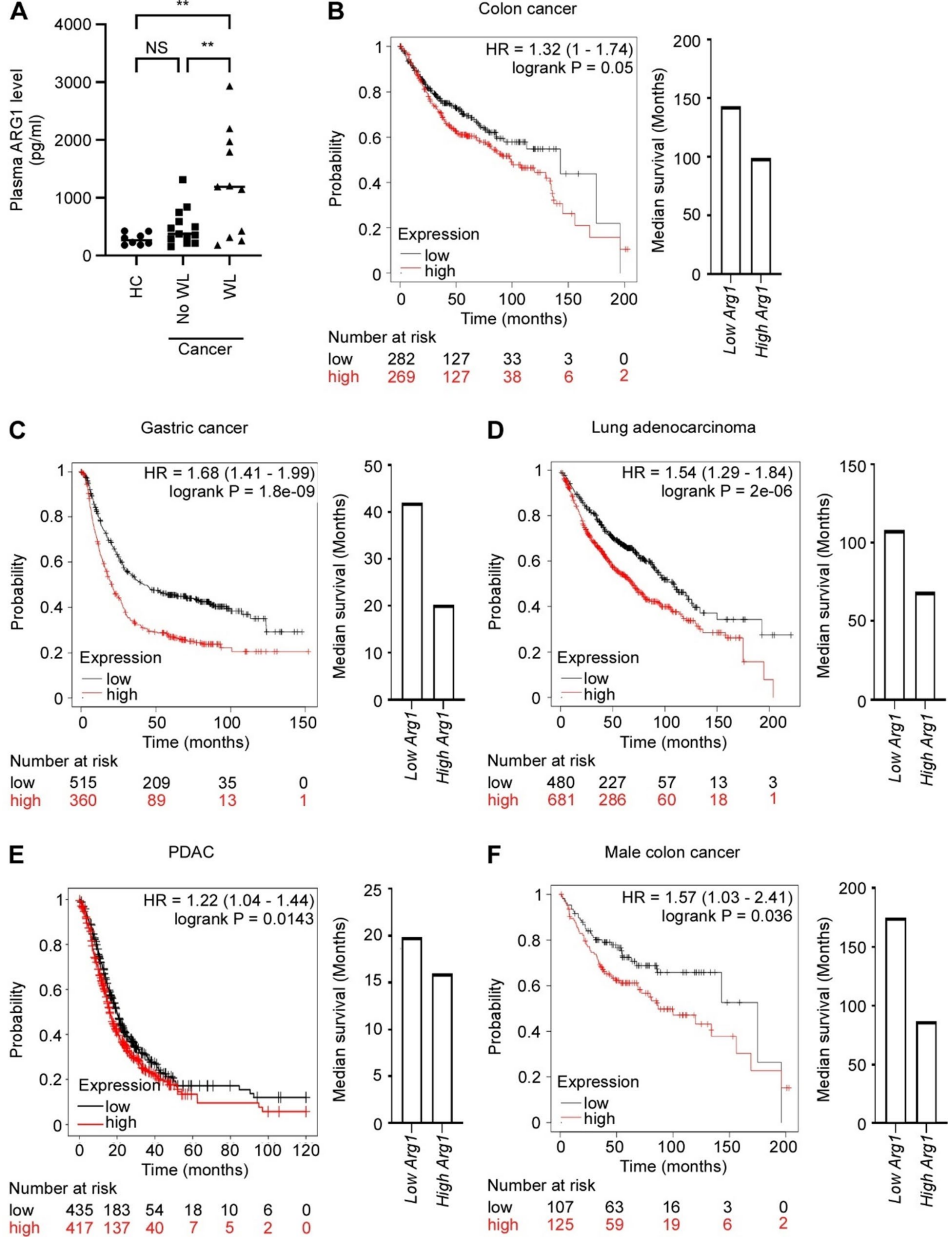
High arginase 1 levels are associated with weight loss and short survival. **A**) ARG1 level in plasma from healthy control subjects (HC, *n* = 8) and cancer patients with > 10% weight loss (WL, *n* = 11) or < 2% WL (*n* = 13), measured by elisa. Statistical significance was evaluated using one-way ANOVA followed by Tukey’s multiple comparisons test. ** *p* < 0.01, NS = not significant. **B-E**) Relationship between *ARG1* gene expression in tumor tissue and overall survival of colon cancer (**B**), gastric cancer (**C**), Lung adenocarcinoma (**D**), Pancreatic ductal adenocarcinoma/PDAC (**E**), and male colon cancer patients (**F**). HR = hazard ratio. The data are obtained using the kmplot.com database tool [29]. High and low expression were defined as described in the method section

Our findings suggest that ARG1-mediated degradation of arginine may contribute to cachexia pathogenesis. To explore whether circulating ARG1 levels are associated with cachexia in patients, we measured ARG1 protein levels in plasma from gastrointestinal and pancreatic cancer patients, two groups at high risk of developing cachexia. While there was no significant difference in ARG1 level between healthy controls (*n* = 8) and non-cachectic cancer patients (*n* = 13), the cancer patients with cachexia (*n* = 11) had significantly elevated plasma ARG1 levels compared to both the control group and the non-cachectic patients (Fig. 5a). Although this does not imply that high ARG1 levels are sufficient to indicate cachexia in individual patients, it supports a potential association between elevated ARG1 and the cachectic state.

Cachexia is associated with limited survival in cancer patients. To further examine the relationship between *ARG1* expression and cachexia, we asked whether increased *ARG1* expression predicts limited survival. Since expansion of neutrophils and MDSCs also is associated with their infiltration into the tumor, we performed meta-analysis of *ARG1* gene expression in tumor biopsies using the Kaplan-Meier plotter database [29]. We analyzed tumors from a selection of cancers where cachexia is frequent: gastric-, colon-, lung- and pancreatic ductal adenocarcinoma (PDAC). For all these cancers, high expression of *ARG1* predicted poorer overall survival with a hazard ration ranging from 1.22 to 1.67 (Fig. 5b-e). The association between *ARG1* expression and survival was not restricted to a particular gender (Fig.S3, Additional file 11), except for colon cancer, where we only found an association in the male patients. For these patients, however, the impact of *ARG1* expression was striking; the median survival time of high *ARG1* expressing patients was 7.3 years (88 months) shorter than the low-expressing patients (Fig. 5f). Together, these findings support an association between elevated arginase 1 levels, cachexia, and poor survival outcomes in cancer.

## Discussion

Knowledge regarding how the complex muscle microenvironment and intercellular interaction within muscle tissue influences muscle mass and function in cachexia, beyond the role of inflammatory mediators remains scarce. Investigating the muscle microenvironmental conditions that drive cachexia may uncover novel therapeutic targets and pave the way for effective interventions.

We find that markers for neutrophils and MDSCs are greatly enhanced in atrophic muscles, suggesting modulation of the muscle immune environment during cachexia. This is in line with recent reports showing that granulocytes and PMN-MDSCs expand in muscles during cachexia and are important for wasting [10, 12]. While the precise mechanisms through which these immune cells disrupt muscle function and contribute to wasting remain largely underexplored, recent studies provide important insights. Olson et al. (2021) suggest that LCN2 produced by granulocytes is important for the modulation of appetite and that by decreasing food intake, LCN2-expressing cells leads to fat and muscle loss [50]. They do not find that LCN2 is important to regulate catabolic processes in muscle tissue. Thus, other effectors are important for this central aspect of cachexia. Dzierlega et al. (2023) showed that activin A released from PMN immune cells was important for muscle loss in cachectic mice. They suggest that wasting may be mediated through the ability of activin A to upregulate E3 ubiquitin mediators in muscle cells [12]. Hayashi et al. (2024) further studied the immune cells associated with cachexia and found that a subset of neutrophil-like monocytes characterized by interleukin 36 gamma (IL36G) production was induced by cachexia and that IL36G was important for muscle loss [13]. Our data suggest that ARG1 from neutrophils and MDSCs contributes to shaping the muscle microenvironment in a manner conducive to cachexia, offering further mechanistic insights into the complex processes that occur in muscles during this debilitating condition.

ARG1 expressing cells can catalyze the conversion of arginine to ornithine, leading to arginine restriction [35]. Restricting arginine has been studied as an immunomodulatory response to impair T cell functions, however, arginine restriction may severely compromise cellular functions also in other cells. We recently showed that arginine restriction compromises mitochondrial functions and accelerates mitophagy in breast cancer cells [42]. Additionally, it has been shown that declining mitochondrial functions in arginine-starved cancer cells are part of a massive metabolic shift, including suppressed transcription of genes involved in oxidative phosphorylation and fragmentation of mitochondria [44]. In muscle cells subjected to arginine restriction, we find that both respiratory capacity and energy production are severely compromised. Interestingly, disturbed mitochondrial functions and respiratory capacity, and declined ATP production in muscle tissue is believed to be early events in the onset of cachexia [51, 52] and studies indicate that the loss of muscle function may precede the onset of visible wasting [52]. If restricted arginine availability underlies these early events, targeting ARG1 could offer new opportunities for developing early interventions that address the onset of cachexia before wasting becomes clinically evident.

While our findings demonstrate clear metabolic impairments in response to arginine restriction, the cellular models used warrant consideration. We observed strong effects in proliferating myoblasts and AC16 cardiomyocytes, and significant, but somewhat more modest effects in differentiated myotubes. Although our myotube cultures were visually confirmed to be predominantly differentiated, we cannot exclude a contribution from residual myoblasts. Similarly, AC16 cells, while expressing several cardiomyocyte markers, are not fully differentiated and may not fully replicate mature cardiac metabolism. Despite these limitations, the consistent sensitivity of these cell types to arginine deprivation suggests that metabolic vulnerability to low arginine is not restricted to one specific differentiation state. Nevertheless, future studies in fully differentiated primary cells and in vivo models are needed to validate and extend these findings and to better define the specific roles of arginine metabolism in mature muscle and heart tissues during cachexia.

Notably, recent studies have shown that myeloid-specific *Arg1* knockout alone is not sufficient to prevent cancer-induced wasting in mice, suggesting that additional factors are likely required to drive the full progression of muscle wasting [12]. This is consistent with the idea that muscle wasting in cachexia is a complex process, influenced by multiple mechanisms, possibly including other non-immune cell and/or immune cell-derived factors such as activin A and IL36G, as recently proposed [12, 13].

Declining mitochondrial health is associated with increased mitophagy [46], and mitophagy, as well as the degradation of other muscle proteins, are suggested to occur in muscles during cachexia. We find that arginine restriction induces autophagy receptors such as SQSTM1, OPTN, and TAX1BP1, which may contribute to protein and mitochondrial degradation in muscles [43]. We also find that the level of GABARAPL1 is increased upon arginine restriction. GABARAPL1 can facilitate autophagy by anchoring autophagy receptors to the autophagosome membrane, and its expression is increased in atrophying muscles in several conditions, including cancer cachexia [53]. Interestingly, although the autophagy proteins are increased in arginine starved skeletal myotubes, the bulk autophagy process do not seem to be accelerated. Rather, in accordance with reduction in mitochondrial markers, selective autophagic degradation of mitochondria may occur. Moreover, the level of STBD1, which is involved in autophagic degradation of glycogen, is greatly enhanced during arginine restriction in muscle cells, suggesting that arginine restriction promotes the selective degradation of glycogen. Glycogen is crucial for maintaining glucose homeostasis [54], and depletion of glycogen has been suggested in muscles during cachexia [55]. However, unraveling whether arginine limitation induces glycogen depletion and its possible contribution to the metabolic shifts that occur in cachexia was beyond the scope of this study.

Similar to observations in skeletal muscle, PMN-like immune cells expand in the heart during cachexia [12]. In arginine restricted cardiomyocytes, we find that mitochondrial functions are severely reduced, and both bulk autophagy and mitophagy is increased. Metabolic deterioration in cardiomyocytes can lead to cardiac dysfunction, and our findings thus suggest that the heart is vulnerable when ARG1-producing immune cells expand. However, while recent evidence from tumor-bearing mice indicates that *Arg1* knockout alone is insufficient to prevent cardiac atrophy [12], it is important to consider that heart function is not solely determined by cardiac mass. Even in the absence of clear atrophy, arginine restriction in the heart may impair cardiac function, compromising its ability to meet the body’s metabolic demands. These functional disruptions could thus contribute to the development of cardiovascular issues in cachexia, independent of structural changes. Further research should investigate the role of arginine restriction in driving these cardiac disruptions and explore potential therapeutic strategies to mitigate its effects.

We find that high ARG1 level is associated with weight loss and shorter survival of cancer patients. This is interesting from a clinical perspective as it suggests that ARG1 could serve as a potential biomarker for disease progression and patient prognosis. Although, this needs to be explored in a larger patient cohort. Moreover, from a therapeutic perspective, a relationship between ARG1-expressing immune cells and declining muscle and cardiac function is interesting. An ARG1 inhibitor is already being tested in several clinical cancer studies as inhibition of ARG1 is suggested to reactivate tumor immunity [44]. In neither of these studies will the effect on muscle mass or function in cachexia patients be assessed. In a similar line, nutritional supplementation of arginine has been considered as a putative therapeutic strategy to boost tumor immune responses. The same strategy could possibly also counteract cachexia. Nevertheless, it should be considered that arginine taken orally is highly susceptible to degradation in the intestine and may not increase blood and tissue arginine levels extensively. The arginine precursor citrulline has been shown to increase blood arginine more efficiently [56]. However, arginine synthesis from citrulline requires ASS1, and muscle lack this enzyme. Whether dietary supplementation of citrulline could efficiently counteract arginine degradation in muscle tissue, leading to a net increase in arginine available for muscle cells requires further study.

Cachexia occurs in patients with diverse illnesses. While it is unclear whether similar underlying mechanisms take place in patients across primary illness, it is interesting that immunological disturbances seem to be a commonality. Moreover, for several of these conditions, immunosuppressive myeloid cells, and MDSCs in particular, has emerged as putative mediators of pathogenesis [57–59]. In several lung diseases, including Chronic obstructive pulmonary disorder, MDSC expansions have been shown [57]. The same is observed in end-stage kidney disease [60]. Patients with either of these conditions are prone to cachexia development [1, 61, 62]. Interestingly, chronic immune activation following virus infection (e.g., HIV and Sars-cov2) is also associated with cachexia [63, 64] and MDSC expansion (that does not necessarily associate to viral load) [58, 65]. Intriguingly, ARG1 levels and activity (as measured by the arginine/ornithine ratio in blood) have been shown to correlate with MDSC in Covid-19 patients [66]. Although this led to plasma L-arginine shortage [66], whether also muscle cells experience arginine restriction in these patients is undetermined. Even without an underlying illness, cachexia can occur in non-human primates exposed to acute radiation, with skeletal muscle loss reaching up to 50% [67]. Conventional fractionated radiotherapy has also been shown to increase the level of MDSCs [68]. Studies that address whether expansion of MDSCs and arginine restriction is directly connected to muscle loss and loss of muscle function in each of these conditions are needed to conclude if common mechanisms can occur. Our study of cancer cachexia proposes a novel mechanism for cachexia and emphasize the need to further explore the role of ARG1-producing myeloid cells in cachexia pathogenesis, as this may pave the way for future therapeutic advancements.

## Conclusions

Our findings identify a potential mechanism underlying cachexia. This mechanism involves the expansion of ARG1-expressing myeloid cells and the localized depletion of arginine, which leads to a reduction in muscle mitochondrial capacity and an increase in catabolic activity.

## Electronic supplementary material

Below is the link to the electronic supplementary material.

Supplementary Material 1

Supplementary Material 2

Supplementary Material 3

Supplementary Material 4

Supplementary Material 5

Supplementary Material 6

Supplementary Material 7

Supplementary Material 8

Supplementary Material 9

Supplementary Material 10

Supplementary Material 11

## Data Availability

The mass spectrometry proteomics data have been deposited to the ProteomeXchange Consortium via the PRIDE [69] partner repository with the dataset identifier PXD060117. All other data in this article can be requested.

## Abbreviations

ARG1: Arginase-1
ASS1: Arginosuccinate synthase 1
ATG8: Autophagy related 8
BafA1: Bafilomycin A1
BP: Biological process
Cacna1i: Calcium Voltage-Gated Channel Subunit Alpha1 I
Chil3/4: Chitinase-like protein 3/4
Cd177: Cluster of differentiation 177 antigen
Cd300lf: CD300 Molecule Like Family Member F
Clec4e: C-type lectin domain family 4 member E
COX: VIII Cytochrome c oxidase subunit 8
CSf3r: Colony stimulating factor 3 receptor
Cxcl13: C-X-C- ligand 13
Cxcr1/2: C-X-C chemokine receptor 1/2
DEG: Differentially expressed genes
Doc2b: Double C2-like domain-containing protein beta
Entpd3: Ectonucleoside triphosphate diphosphohydrolase 3
FC: Fold change
FDA: Food and Drug Administration
FDR: False discovery rate
Fpr1: Formyl peptide receptor 1
GABARAPL1: Gamma-aminobutyric acid receptor-associated protein-like 1
GO: Gene ontology
Gypa: Glycophorin A
HC: Healthy control
HR: Hazard ratio
Il1b: Interleukin-1 beta
Il1f9: Interleukin-1 family member 9
IL36G: Interleukin 36 gamma
Itgb2l: Integrin beta 2-like
LC-MS/MS: Liquid Chromatography with tandem mass spectrometry
Lcn2: Lipocalin-2
LDH: Lactate dehydrogenase
LLC: Lewis lung carcinoma
Ltf: Lactotransferrin
Ly6G: Lymphocyte antigen 6G
MDSC: Myeloid-derived suppressor cell
Mmp8: Matrix metalloproteinase-8
Mrgpra2b: MAS-related GPR, member A2B
Mt2: Metallothionein-2
Ngp: Neutrophilic granule protein
NS: Not significant
OCR: Oxygen consumption rate
OGJ: Oesophagogastric junctional adenocarcinoma
OPTN: Optineurin
OS: Overall survival
PCA: Principal component analysis
PDAC: Pancreatic ductal adenocarcinoma
PMN-MDSC: Polymorphonuclear myeloid-derived suppressor cell
R: Arginine
Retnlg: Resistin-like gamma
S100A8/9: S100 calcium-binding protein A8/9
Sell: Selectin L
SEM: Standard error of the mean
Slc: Solute carrier
SQSTM1: Sequestosome 1
STBD1: Starch-binding domain-containing protein 1
Stfa2l1: Stefin A2 like 1
TAX1BP1: Tax1 binding protein 1
TFAM: Mitochondrial transcription factor A
TFB1M: Dimethyladenosine transferase 1, mitochondrial
TIMM23: Translocase of inner mitochondrial membrane 23
Trem1/3: Triggering receptor expressed on myeloid cells 1/3
WL: Weight loss
