# Correction: A role of arginase-1-expressing myeloid cells in cachexia

**DOI:** 10.1186/s40170-025-00412-3

**Published:** 2025-11-07

**Authors:** Apsana Lamsal, Sonja Benedikte Andersen, Unni Nonstad, Natalie Jayne Kurganovs, Richard JE Skipworth, Geir Bjørkøy, Kristine Pettersen

**Affiliations:** 1 Department of Biomedical Laboratory Science, Faculty of Natural Sciences, Norwegian University of Science and Technology, Trondheim, Norway; 2 Centre of Molecular Inflammation Research, Department of Cancer Research and Molecular Medicine, Faculty of Medicine and Health Sciences, Norwegian University of Science and Technology, Trondheim, Norway; 3 Department of Molecular Cell Biology, Institute for Cancer Research, Oslo University Hospital, Montebello, Oslo Norway; 4 Centre for Cancer Cell Reprogramming, Faculty of Medicine, University of Oslo, Oslo, Norway; 5 Institute for Cancer Research, Department of Tumor Biology, Oslo University Hospital, Montebello, Oslo Norway; 6 Clinical Surgery, University of Edinburgh, Royal Infirmary of Edinburgh, Edinburgh, Scotland

**Correction to: Cancer % Metabolism (2025) 13:27**

10.1186/s40170-025-00396-0

The original publication of this article contained incorrect author names. The incorrect and correct information is listed in this correction article. The original article has been updated.

Incorrect

Skipworth Richard JE

Lamsal Apsana

Andersen Sonja Benedikte

Nonstad Unni

Kurganovs Natalie Jayne

Bjørkøy Geir 

Pettersen Kristine

Correct

Richard JE Skipworth 

Apsana Lamsal

Sonja Benedikte Andersen

Unni Nonstad

Natalie Jayne Kurganovs

Geir Bjørkøy

Kristine Pettersen

