# Supplementary material for: A role of arginase-1-expressing myeloid cells in cachexia
Source: Cancer Metab. 2025 Jun 5;13:27. doi: 10.1186/s40170-025-00396-0 (PMC12142917; doi:10.1186/s40170-025-00396-0)
Supplement: Supplementary file 1 — Supplementary Material 1 [file 40170_2025_396_MOESM1_ESM.pdf]

Table S1. Demographics of patients from the Edinburgh Cancer Cachexia Cohort (ECCC). OGJ= Oesophagogastric junctional adenocarcinoma, *Weight loss during the last 6 months prior to sampling.

**Clinical characteristics**		**Control (n=8)**	**Weight stable Cancer (n=13)**	**Weight losing Cancer (n=11)**
**Age; median (range)**		76 (36-78)	70 (56-83)	71 (48-81)
**Sex**	Female	4	2	6
	Male	4	10	5
**Cancer type**	OGJ		2	1
	Oesophagus		7	3
	Pancreas		2	3
	Gastric		2	4
**Pre-illness BMI (kg/m^2); mean (SD)**		21.4 (1.7)	22.4 (1.8)	22.3 (2.3)
**BMI (kg/m2); mean (SD)**		21.4 (1.7)	22.5 (1.7)	18.6 (2.1)
**% Weight loss***		0	-0.5 (2.4)	16.6 (5.6)
**mid-arm muscle circumference (MAMC); mean (SD)**		22.5 (2.5)	23.7 (2.2)	21.1 (1.6)
**Triceps skinfold (TSF) thickness; mean (SD)**		12.2 (3.7)	11 (3)	10 (4)
**CRP (mg/L); mean (SD)**		1 (0.5)	6.7 (6.6)	21.5 (29.1)
