# Supplementary material for: A role of arginase-1-expressing myeloid cells in cachexia
Source: Cancer Metab. 2025 Jun 5;13:27. doi: 10.1186/s40170-025-00396-0 (PMC12142917; doi:10.1186/s40170-025-00396-0)
Supplement: Supplementary file 2 — Supplementary Material 2 [file 40170_2025_396_MOESM2_ESM.pdf]

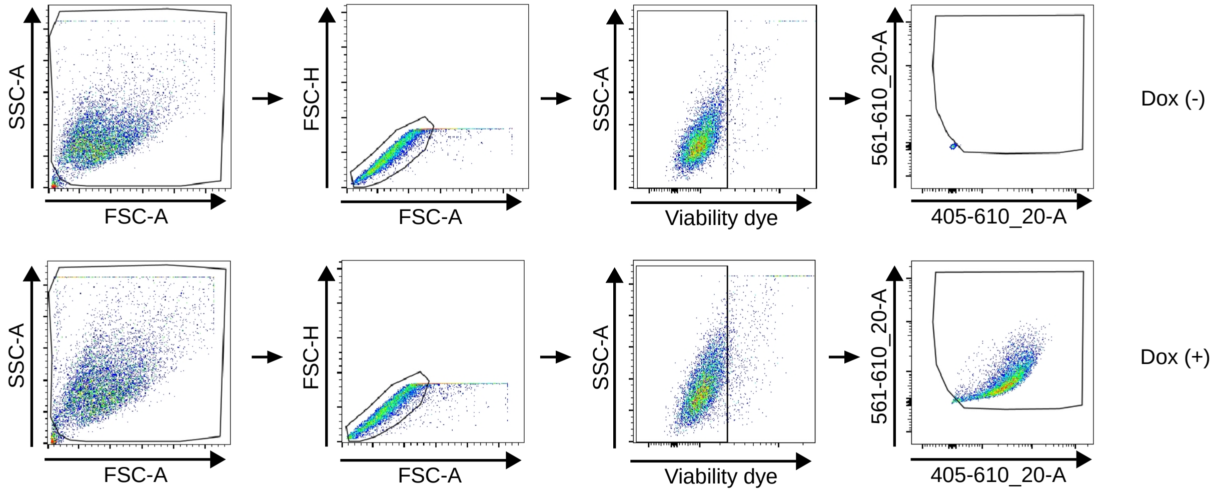

SSC-A
FSC-A
FSC-H
FSC-A
SSC-A
Viability dye
561-610_20-A
405-610_20-A
Dox (-)
SSC-A
FSC-A
FSC-H
FSC-A
SSC-A
Viability dye
561-610_20-A
405-610_20-A
Dox (+)

Figure S1. Gating strategy for mitophagy flux determination

Gating strategy for experiments shown in Fig. 4G-H.
