# Supplementary material for: A role of arginase-1-expressing myeloid cells in cachexia
Source: Cancer Metab. 2025 Jun 5;13:27. doi: 10.1186/s40170-025-00396-0 (PMC12142917; doi:10.1186/s40170-025-00396-0)
Supplement: Supplementary file 10 — Supplementary Material 10 [file 40170_2025_396_MOESM10_ESM.pdf]

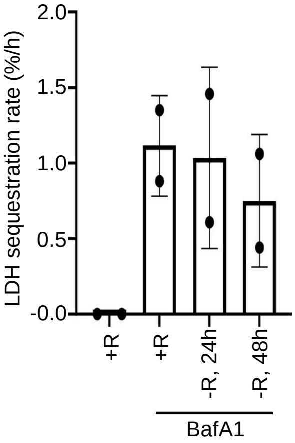

LDH sequestration rate (%/h)
2.0
1.5
1.0
0.5
-0.0
+R
+R
-R, 24h
-R, 48h
BafA1

Figure S2. Bulk autophagy is not increased in C2C12 myotubes. Lactate dehydrogenase (LDH) sequestration determined in C2C12 myotubes grown in medium with arginine (400µM) or without arginine (+R and -R, respectively), +/-Bafilomycin A1 (BafA1, 100nM) for 24h or 48h as indicated, using an LDH sequestration assay (Referenced in main paper) (n=2).
