# Supplementary material for: A role of arginase-1-expressing myeloid cells in cachexia
Source: Cancer Metab. 2025 Jun 5;13:27. doi: 10.1186/s40170-025-00396-0 (PMC12142917; doi:10.1186/s40170-025-00396-0)
Supplement: Supplementary file 11 — Supplementary Material 11 [file 40170_2025_396_MOESM11_ESM.pdf]

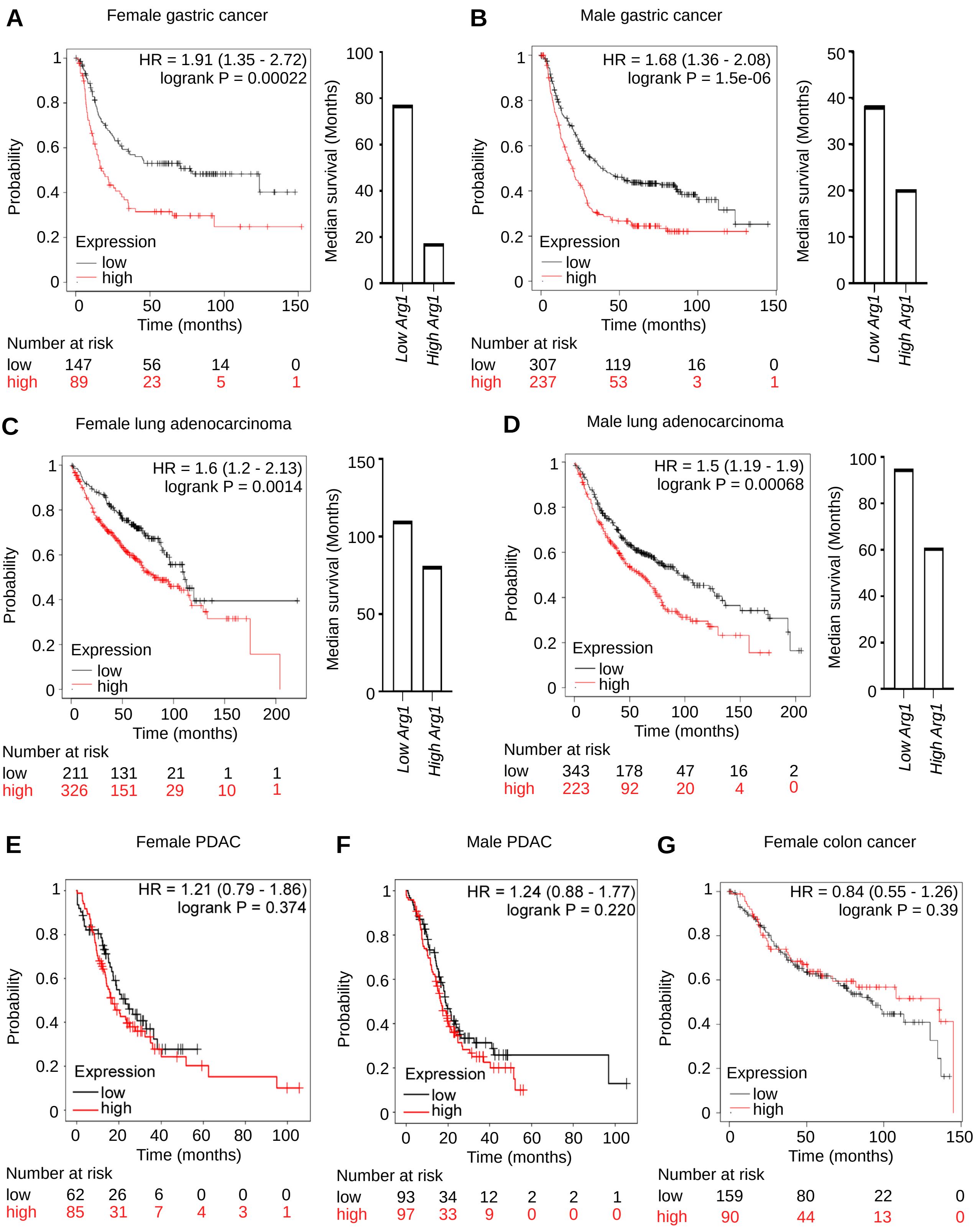
A
Female gastric cancer
HR = 1.91 (1.35 - 2.72)
logrank P = 0.00022
Probability
Time (months)
Expression
low
high
Number at risk
low 147 56 14 0
high 89 23 5 1
Median survival (Months)
Low Arg1
High Arg1
B
Male gastric cancer
HR = 1.68 (1.36 - 2.08)
logrank P = 1.5e-06
Probability
Time (months)
Expression
low
high
Number at risk
low 307 119 16 0
high 237 53 3 1
Median survival (Months)
Low Arg1
High Arg1
C
Female lung adenocarcinoma
HR = 1.6 (1.2 - 2.13)
logrank P = 0.0014
Probability
Time (months)
Expression
low
high
Number at risk
low 211 131 21 1 1
high 326 151 29 10 1
Median survival (Months)
Low Arg1
High Arg1
D
Male lung adenocarcinoma
HR = 1.5 (1.19 - 1.9)
logrank P = 0.00068
Probability
Time (months)
Expression
low
high
Number at risk
low 343 178 47 16 2
high 223 92 20 4 0
Median survival (Months)
Low Arg1
High Arg1
E
Female PDAC
HR = 1.21 (0.79 - 1.86)
logrank P = 0.374
Probability
Time (months)
Expression
low
high
Number at risk
low 62 26 6 0 0 0
high 85 31 7 4 3 1
F
Male PDAC
HR = 1.24 (0.88 - 1.77)
logrank P = 0.220
Probability
Time (months)
Expression
low
high
Number at risk
low 93 34 12 2 2 1
high 97 33 9 0 0 0
G
Female colon cancer
HR = 0.84 (0.55 - 1.26)
logrank P = 0.39
Probability
Time (months)
Expression
low
high
Number at risk
low 159 80 22 0
high 90 44 13 0

Figure S3. Association between tumor Arg1 gene expression and survival. Relationship between Arg1 gene expression in tumor tissue and overall survival of female (A) and male (B) gastric cancer, female (C) and male (D) lung adenocarcinoma, female (E) and male (F) Pancreatic ductal adenocarcinoma/ PDAC, and female colon cancer patients (G). HR= hazard ratio. The data are obtained using the kmplot.com database tool (referenced in the main paper). High and low expression were defined as described in the method section.
